# Assessment of Sinkholes Investigations in Jangseong-Gun Area, South Korea, and Recommendations for Similar Studies

**DOI:** 10.3390/ijerph19031111

**Published:** 2022-01-20

**Authors:** Khaqan Baluch, Jung-Gyu Kim, Jong-Gwan Kim, Young Hun Ko, Seung-Won Jung, Sher Q. Baluch

**Affiliations:** 1 Department of Mineral Resources and Energy Engineering, Jeonbuk National University, Deokjin-gu, Jeonju 54896, Korea; khaqan_baluch@hotmail.co.uk; 2 Department of Energy and Resources Engineering, Chonnam National University, 77 Yongbong-ro, Buk-gu, Gwangju 61186, Korea; evangelong@hanmail.net; 3 Department of Geotechnical Engineering Research, Korea Institute of Civil Engineering and Building Technology, 283 Goyang-daero, Ilsanseo-gu, Goyang 10223, Korea; younghunko@kict.re.kr; 4 Guns and Explosives Safety Technology Association, 50, Mapo-daero 1-gil, Mapo-gu, Seoul 04162, Korea; wjd071722@naver.com; 5 Consulting Engineer, EDACS International, 6000 Ohrid, North Macedonia

**Keywords:** sinkholes, geophysical and geotechnical investigation, impact of mining and agriculture on groundwater regime, karst cavities, borehole image processing system, limestone mining, interpretation of satellite imagery

## Abstract

This paper reviews the site investigation field data and access work performed between 2016 and 2019 in the study area located close to Gun-dong mine. The research was aimed at defining the cause of sinkholes and their relationship with the underlying karstic limestone bedrock and nearby mining activities. Only a limited number of small sinkholes appeared in 2014, 2016, and 2018 in the agricultural land close to the limestone mine. The previously open pit mine started its underground operations in 2007. Since then, the mine has developed, and is now comprised of, large underground excavations at several levels below the surface. The studies carried out concluded that the appearance of sinkholes may be related to a general lowering of the groundwater table because of nearby agricultural and mining activities and also due to over-extraction of water due to increased urban use. Whilst these are the best determinations, this paper identifies missing elements of the previous investigations mentioned above, some issues with the interpretation of poorly prepared borehole logs and the improper preservation of borehole cores. The authors make recommendations for a systematic approach for implementation of an investigation strategy. This paper concludes that the appearance of sinkholes is a natural phenomenon, developing over geological time. However, human intervention contributes to sinkhole formation, which in urban areas may result in human, property, and economic losses. A better understanding, based on a methodical approach and suitable technologies, can determine the causes of sinkholes and can lead to the formulation of solutions and the implementation of economically and socially acceptable mitigation measures.

## 1. Introduction

A sinkhole is usually defined as a collapse of sediments overlying bedrock (overburden) or a depression in the ground formed as result of soil migration or leaching into underlying rock mass containing open fractures or cavities formed naturally due to karst development in carbonate rocks, such as limestone and dolomite, or due to mining activity. Collapse of open voids in rock also results in the formation of sinkholes [1]. Sinkhole occurrence is a global phenomenon associated with geological processes. Large sinkholes and subsidence can impact the topography of the region [2], the groundwater regime, cave formation, and create problems for the human habitat, such as buildings, roads, and other infrastructure installations. South Korea has experienced infrastructure damage due to sinkholes [3].

### Classification of Sinkholes

**Figure 1:**
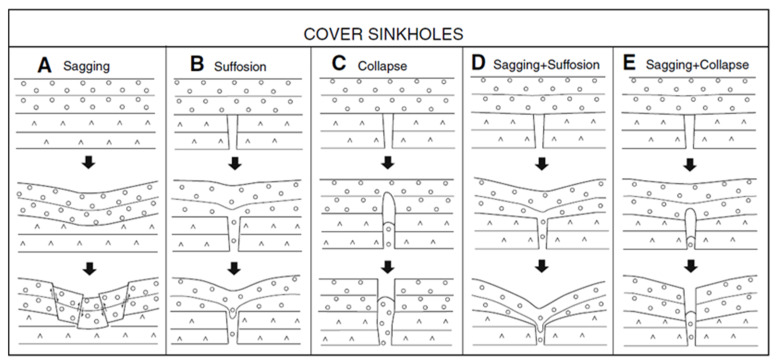
Diagram showing the main subsidence mechanisms involved in the generation of cover sinkholes.

There are six main types of sinkholes, classified in two categories [4,5]. A generic classification of sinkholes is given in Figure 1 and described thereafter.

- Sinkholes in soil or overburden: These are further divided into (a) suffusion sinkholes and (b) dropout sinkholes. Suffusion and dropout sinkholes form entirely within the soil profile. These are caused by water infiltration, resulting in washing non-cohesive soil down into open cavities or discontinuities in the rockhead. Figure 1 shows the different types of sinkholes.
- Sinkholes within bedrock: the formation of dissolution, collapse, caprock, and buried sinkholes is dependent on erosion processes occurring over geological time. They are related to the solubility and other properties, mostly, of calcareous rocks. In addition to the multiple natural processes, sinkhole formation is also the result of human interference, such as active or disused mining, tunneling, and sewage water supply works.

## 2. Case Study of Sinkholes Investigations in Jangseong-gun Area, South Korea

The appearance of sinkholes in agricultural land close to a limestone mining area prompted fears that local infrastructure, such as a highspeed railway embankment and farmland, could be in danger. A series of investigations were carried out [6,7,8,9] and a non-dispersive anti-washout grout was developed to fill the underground cavities [10] if warranted. These investigations’ data and results were reviewed, using this as a case study to assess the suitability of the investigation methods, interpretation of data collected, and validity of the conclusions reached.

### 2.1. Description of the Study Area

**Figure 2:**
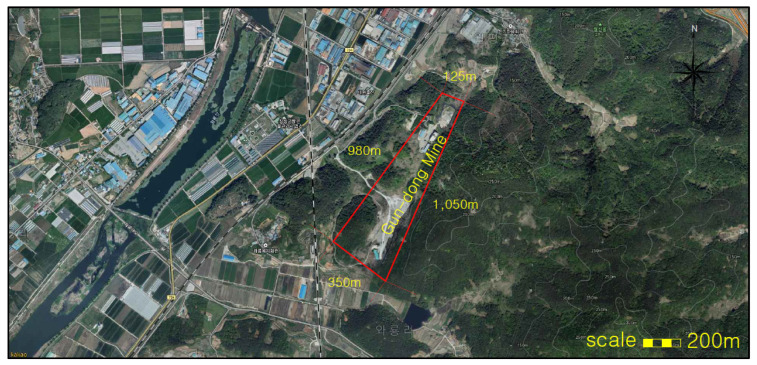
Aerial view of Gun-dong mine and adjacent areas.

**Figure 3:**
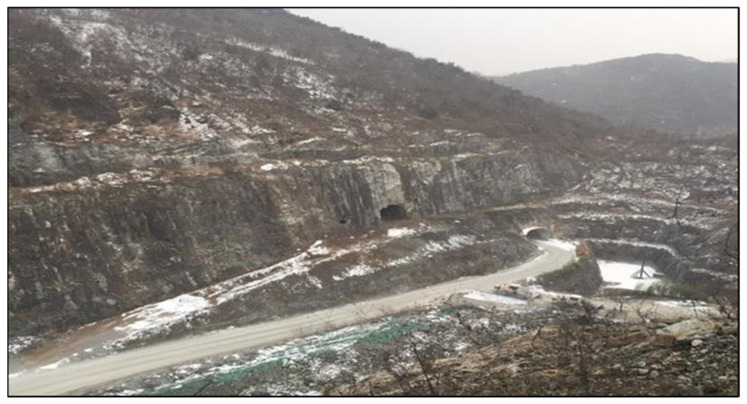
A view of the Gun-dong mine entrance and storage reservoir.

The study area (E: 126°45′10″ and N: 35°15′17″), as shown in Figure 2, is in Jangseong-gun of Jeollanam-do province. There are also three other mines within a 2 km radius, all mostly extracting low-grade limestone for cement manufacture. The nearest to Gun-dong mine (Jangseong-gun Mine as locally known) started its life as an open pit mine, until 2007 when it was partially backfilled with waste material and then underground mining commenced. As of 2019, the 11th, 12th, and 14th levels were being mined, with the lowest 14th level being at −220 m asl. An aerial view of Gun-dong mine is given in Figure 2, whereas Figure 3 shows the entrance and mine water reservoir.

**Figure 4:**
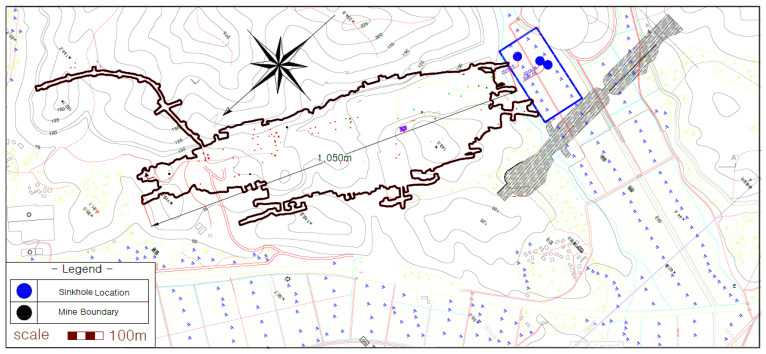
Gun-dong mine layout and adjacent area of sinkholes.

**Figure 5:**
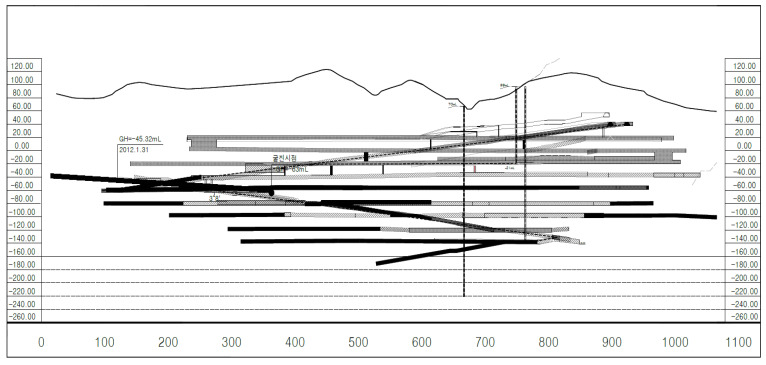
Developed longitudinal section of Gun-dong mine along the north-south direction showing various mine excavation levels as of 2018. Black and grey pattern used are to create perspective showing lateral and vertical distribution of various mine levels.

The total areal extent of Gun-dong Mine and its proximity to sinkholes is shown in Figure 4. The mining is performed by open plan excavation of large 3–4 corridors at each floor level, leaving the required rock pillars for support. Inclined access ramps reach the various mine levels. The average length of the mine is 900 m and the maximum width of each level is approximately 200 m, as each mining section is standardized with an average height of 6 m for each excavated section, 12 m for pillar width, and 14 m between mining levels. A developed cross section of the mine is shown in Figure 5.

### 2.2. History of Sinkholes Development in Waryong-ri and Jangseong-gun Area

**Figure 6:**
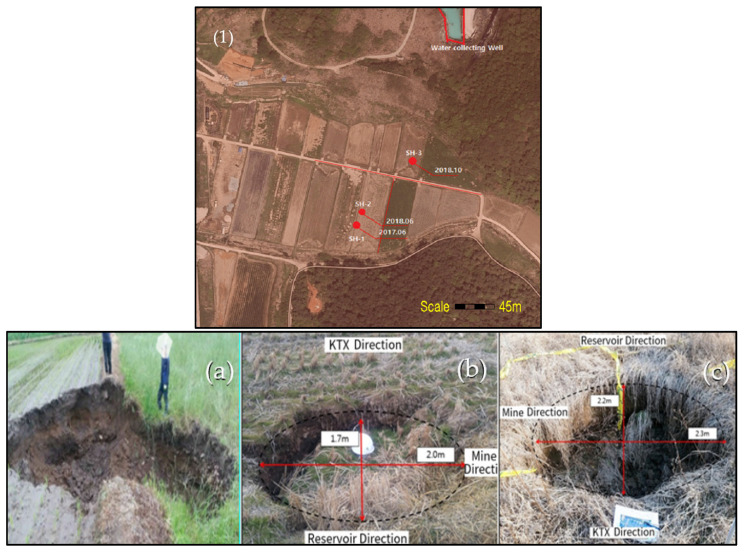
Sinkhole locations and close-up photographs of sinkholes in agricultural land adjacent to Gun-dong mine, (1) Sinkhole locations, (**a**) 2017.06 (Sinkhole-1), (**b**) 2018.06 (Sinkhole-2), (**c**) 2018.10 (Sinkhole-3).

The periodic appearance of sinkholes goes back to before 2014. Recent ground subsidence occurred in the farmland in the Jangseong-gun area, three times in the last three years (Jun. 2017, Jun. 2018, Oct. 2018) within the land parcels 36-18, 36-19, and 35-21, raising fears about the acceleration in the incidence of sinkholes in the area. The recent sinkholes were of small scale and relatively shallow, with largest being in the land parcel 36-18. There has been no survey record of these sinkholes, and these were backfilled, apparently without monitoring their activity; i.e., development into larger and deeper sinkholes. The location and photographs of the sinkholes are given in Figure 6. 

The appearance of sinkholes prompted an investigation along the highspeed railway, due to embankment safety concerns. Further investigations were undertaken after the owners of the agricultural land raised concerns related to the appearance of sinkholes due to the ongoing mining activity nearby. Geophysical and geotechnical investigations [11,12,13] were performed, which are summarized hereafter.

### 2.3. Summary of Investigations Performed

The investigations were conducted over a period, by various institutions. The following investigation reports and publicly available data on geology, hydrogeology, and topography were studied for the purpose of this research:

- Chonnam national university, in 2008, comprised of electrical resistivity for the detection of underground cavities in relation to the first appearance of subsidence in the area.
- Korea Railroad Research Institute, in 2014, comprised of electrical resistivity survey and drillings.
- Investigations by Chosun University, in 2016, comprised of electrical resistivity.
- Chonnam national university, in 2020, comprised of electrical resistivity, hydrogeology, and topography of the study area. (Korea Cement Research Report).

The lead author was directly involved in some of the field investigations and data collection and processing. The investigations performed are listed as follows:

#### 2.3.1. Previous Investigations Performed by Various Institutions

Different methods of investigation were used based on the site conditions and accessibility [14,15,16,17,18]. Full details of the previous investigations are given in Section 3, and the investigation data and results are analyzed and commented upon in the context of sinkhole development in the study area. A list of the various investigations is summarized below:

- Geophysical investigations
- Borehole drilling investigation
- Borehole imaging (BIPS) and epidemiological investigation
- Determination of physical and mechanical properties of the rock mass
- Numerical analysis and review of ground subsidence

#### 2.3.2. Investigation and Research Performed by the Authors

The authors noted gaps in the investigations carried out investigating sinkholes in the Gun-dong mine area, South Korea, and this caused a certain loss of important field data.

- Surface Geological, tectonic, and topographic Assessment
- Participation and data collection during field investigations
- Hydrogeological investigations
- Evaluation of the maximum settlement amount and the range of settlement effects by the distribution pattern of the mining area
- Analysis of settlement of agricultural land near Waryong-ri, due to the excavation of the Gundong mine

### 2.4. Geomorphological, Geological, and Tectonic Structures

**Figure 7:**
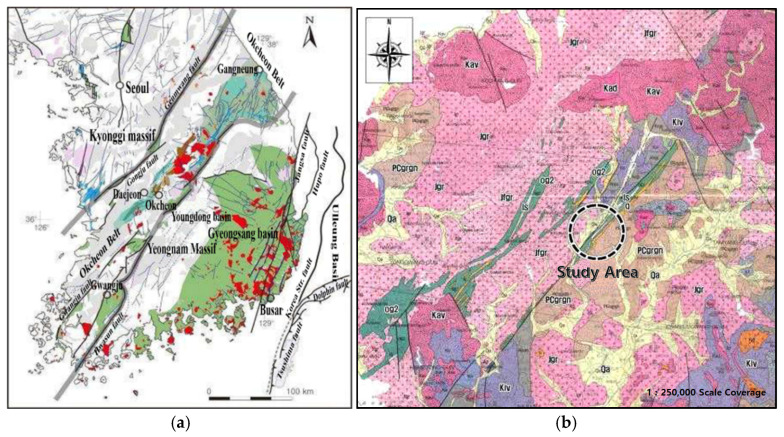
Regional structural and geological maps of the study area, (**a**) regional structural and tectonic map, (**b**) regional geological map of area.

The study area is underlain by a Sobaeksan granite gneiss complex belonging to the Precambrian and Ogcheon formation group, comprised of lower phyllites, limestone, quartzite traversed by Jurassic Mesozoic foliated granites, hornblende, and granites. In addition, the Jinan strata (sandstones and mudstones) of the contemporary Cretaceous period appear in unconformable contact. The Quaternary alluvium of the Cenozoic Era overlies the bedrock in various thickness. Regionally, the sedimentation process is ongoing along rivers and valleys. Regional geological and tectonic maps of the area are shown in Figure 7 [19]. 

**Figure 8:**
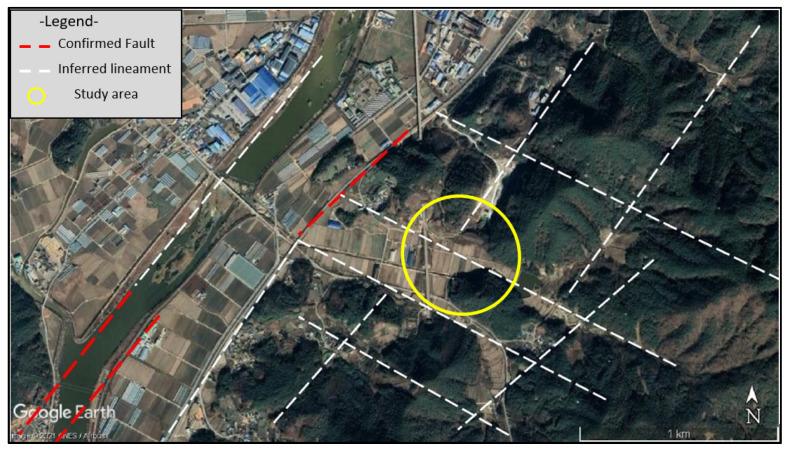
Regional lineaments in the vicinity of the Gun-dong Mine area.

The regional tectonic structure shown (Figure 7) is dominated by linear features, trending NW to SE. These are the controlling tectonic features, such as faults and major joints in the bedrock. The same trend is reflected in the lithological distribution and unconformities, creating long valley profiles and structurally controlling the Hwangr-yong-gang River alignment in the region near Jangseong, as it flows through Imgok toward Songjeong. To complete this research, the authors decided to analyze the regional tectonic structure of the region and in the sinkhole area. The known faults and major joint sets are shown in red, regional faults and structurally controlling lineaments were identified using google satellite images. These interpreted lineaments are approximately marked as straight dashed lines, as shown (Figure 8). Slight variations in alignment and direction, including slight curvature and displacements, may occur locally.

There is topographical evidence of fault displacement due to tectonic activity, and this may have contributed to the formation of shear and fracture zones, leading to the formation of karst. In addition, there are east–west trending tectonic joints, which probably act as conduits for groundwater flow towards the river. As such the river and its alluvium act as a regional drainage channel for surface and groundwater flow.

**Figure 9:**
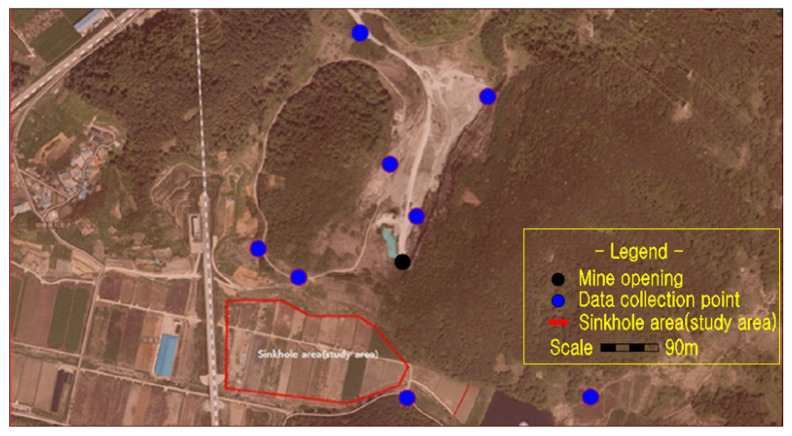
Location of discontinuities data collection points.

**Figure 10:**
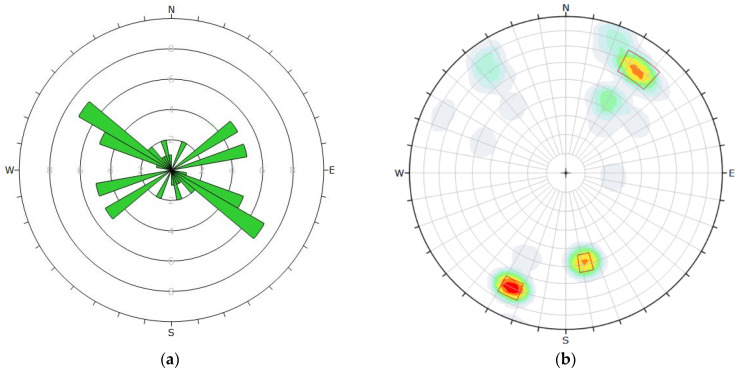
Poles and apparent strikes of major discontinuities, (**a**) rosette preset view, (**b**) polar view.

A discontinuities survey was carried out by one of the researchers for this paper in 2021, with 33 measurements in the area. The location of these measurement is indicated in Figure 9. As shown below in the DIPS rosette diagram, this largely confirmed the interpretation of regional lineaments. Discontinuities data are analyzed in Figure 10.

**Figure 11:**
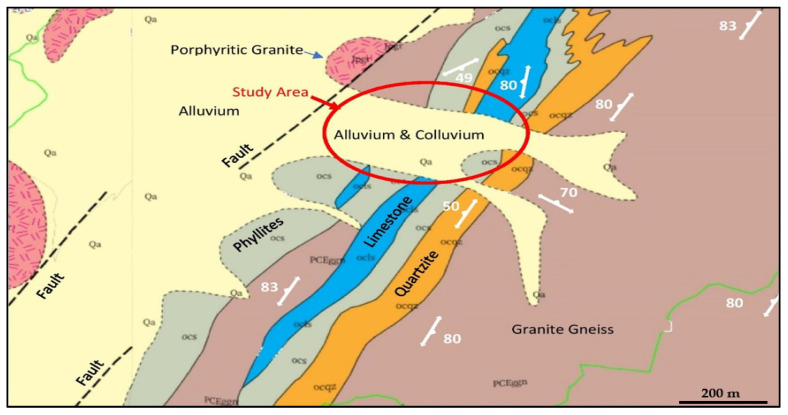
Site geological map of the study area.

The site geological map (Figure 11) shows three sets of main discontinuities, largely perpendicular to each other (Lineament 1: N117E to N125E, Lineament 2: N355W to N020NE: Lineament 3: N065E to N080E). In the borehole logs, NBH-1 to NBH-4, the same three major sets are repeatedly encountered, with inclination range between 45° and 80° with the majority in the range between 60° and 80°. The bedding joints dip between 20° and 40°, averaging at 30°. Some of the joints have slickensides on the surface. Based on the boreholes investigations, the stratigraphic structure is summarized in Table 1.

The limestone formation containing Okcheondae limestone (Okcheon Formation) is known to be karstic in nature and is responsible for the subsidence in Muan (34′59.7° N, 126°29′ E) area [20], located about 35 km from the study area. This limestone spans (Figure 11) from Gangwon area to Chungbuk–Chungnam, and Jeollabuk-do–Jeollanam-do. The same limestone horizons occur along the Songjeong line of Gwangju, in a northeast to southwest direction. The limestone is traversed by gneisses [19].

The geology of the study area shows bedrock containing schists and limestone. The schists include black phyllite, limestone, and gray-green schist. 

#### Gun-Dong Mine Limestone Horizon

The low-grade limestone horizon being mined is used in the manufacture of cement and has an average thickness of about 200 m and average width of 400 m. The limestone is fine to coarse grained and has a relatively pure milky white color, which changes gradually to schist in the strike direction, according to Korea Resources Corporation, 2006 [21]. In some areas there are unconformities, where gneisses cross the limestone formation. 

Limestone generally contains almost 70–90% of the carbonate minerals present in the form of calcite, and the dolomite is composed of mostly gray to dark gray and some white to grayish white crystals. The general mineral composition is given in Table 2.

The limestone composition with a large percentage of sericite suggests that it has undergone significant hydrothermal alteration. Aggressive water flow through fractures or joints dissolves the surrounding limestone rock mass, enlarging these discontinuities, which in some cases leads to the eventual formation of karst or open fissures.

## 3. Description and Results of Geophysical Investigations

The geophysical explorations were carried out separately by three different institutions, and these are as follows:

1. Investigations by Chosun University in 2016 comprised electrical resistivity. The geophysical traverses are marked by Line-1, Line-2, Line-3, Line-4, and Line-5.
2. The Korea Railroad Research Institute in 2014 carried out an electricity resistivity exploration to analyze and determine the blast effects of the mine on the KTX line, research report. The geophysical traverses are marked by Lines 1 to 5.
3. Chonnam National University Overseas Resource Development Research Center in 2008, carried out investigations using electrical resistivity methods to investigate the first small subsidence over a large area observed in the area.

Some of the exploration lines were overlapped, in order to obtain a better idea of the cavities present in the subsurface. The most recent survey by Chosun university ameliorated the observed errors in previous surveys conducted by Chonnam national university and Korea Railroad Research Institute. The data from various lines were combined to improve the analysis and this resulted in a better resolution. In addition, Chosun University extended the length of the exploration lines, up to 150 m through the KTX line, electrode distance was kept at 30 m, and they measured the electrode number n value up to 10, compared with Korea Railroad Research Institute, which measured up to n = 8 and used relatively small electrode intervals of 20 m. 

**Figure 12:**
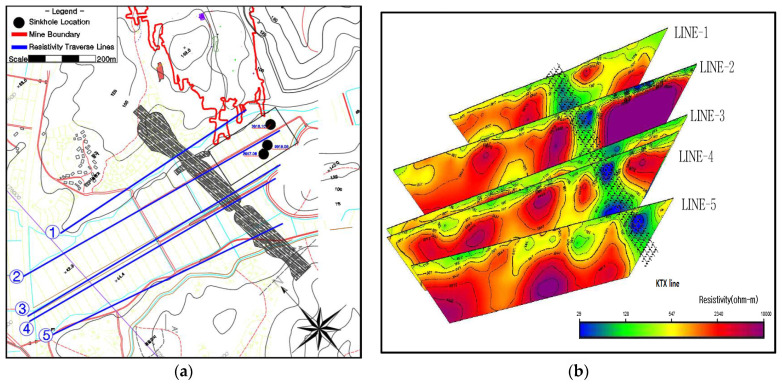
(**a**) Chosun University resistivity traverse lines in relation to the mine footprint and sinkholes (**b**) resistivity data of Chosun University, in railway embankment Area 2016.

A plan of geophysical traverses is given in (a), and an interpretation of resistivity data is given in (b) of Figure 12. When analyzing the investigation conducted by Chosun University, the results show a strong image of a low non-resistance group, which are more reliable than in the Korea Railroad Research Institute’s exploration work. 

Electrical resistivity surveying is an excellent tool for the determination of approximate soil boundaries and aquifer delineation [22]. In conjunction with ground penetrating radar (GPR), it can also be used for detection of clayey infill characterized sinkholes [23]. However, it is not a precise tool for the determination of bedrock characteristics, accurate level of rockhead profile, and karstic cavities within relatively sound limestone. Instead of the electrical resistivity method, seismic refraction surveying with a high energy source and optimum spacing of geophones provides more reliable data [24].

The resistivity profile data interpreted by Chosun University combines data from two studies and the exploration results show that the study area bedrock consists of limestone, and there are several low resistivity areas with enhanced weathering along the regional tectonic faults and major joints and aquifers within the limestone bedrock. 

It appears that the sectional profile of the railway embankment (KTX Line), which is a large above ground structure in the form of an embankment, was not considered in the interpreted resistivity sections, and the ground profile is shown as a near horizontal or sloping. This greatly affected the interpretation results. 

**Figure 13:**
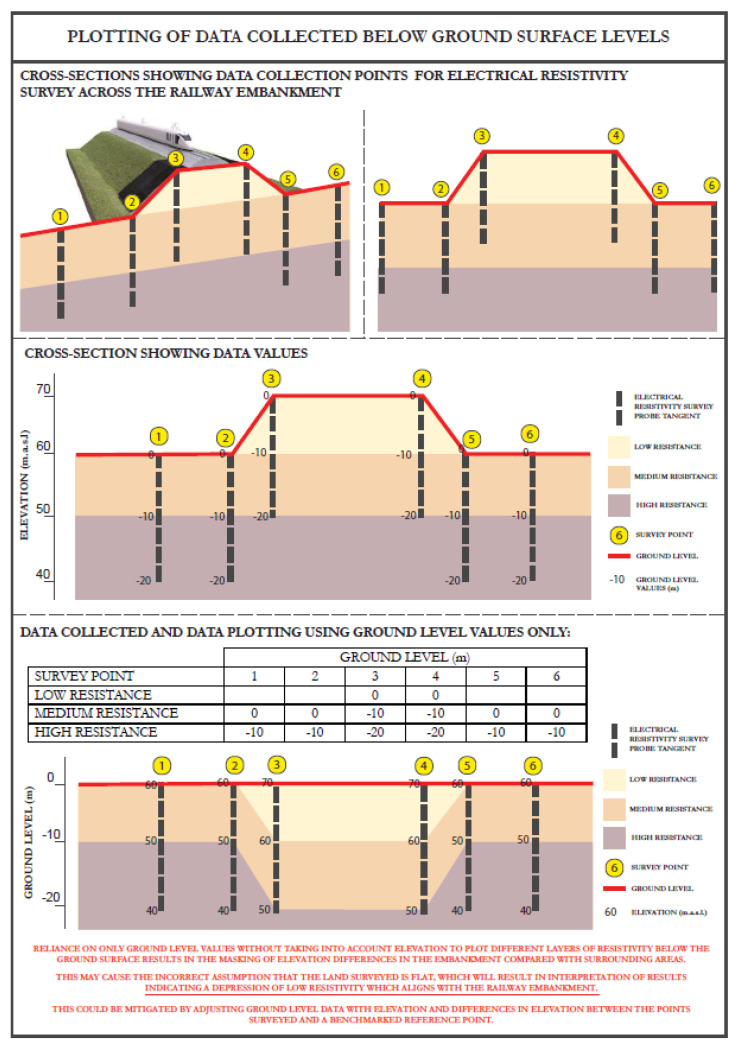
Explanation of apparent depressions below ground surface level, when embankment profile is ignored.

The above issue is further explained in simple terms with the explanation of an apparent distortion and its adjustment, as given in Figure 13, which is self-explanatory. The impact of this distortion may be summarized as follows:

- By not adjusting the resistivity profiles for distortion in the electrode spacing and elevation, the railway embankment appears as a downward low-resistivity corridor (Figure 12b). This is not borne out by the borehole data and the longitudinal geological section along the railway embankment (Figure 19).
- There also seems to be problem whereby the resistivity profiles show potentially large clay-filled cavities and caverns below the rockhead, which normally would not have shown up in a resistivity survey.
- The borehole’s data also do not support the resistivity profile in the same area.

In line with the above comments, the authors have had similar experiences with electrical resistivity surveys elsewhere. In such cases, difficulties were overcome by using additional geophysical tools in conjunction with resistivity profiling. Very low frequency (VLF) equipment is much better suited for the detection of major faults. In addition, the use of ground radar is also valuable for the detection of near surface openings and cavities.

## 4. Description and Results of Borehole Investigations

The purpose of the drilling investigation was to obtain samples of the overburden sediments and the underlying bedrock. The cores recovered from a borehole offered an opportunity for direct inspection of fracture zones and their filling materials along the borehole axis. The selected samples were tested for compressive strength and other relevant properties. 

**Figure 14:**
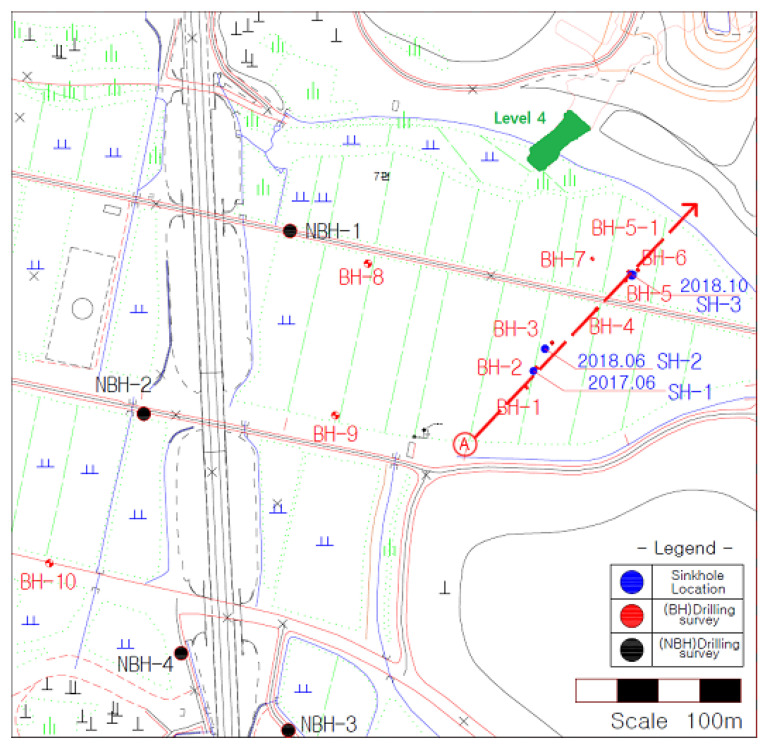
Location of drilling investigations in the study area.

In the first drilling campaign, Boreholes NBH-1 to NBH-4 were drilled along the railway embankment. Another line of BH1 to BH5 was drilled roughly along the line of sinkholes. In addition, BH5-1 as a replacement hole and another borehole (BH6) close to Sinkhole-3 were drilled. Additional boreholes (BH7, BH8, BH9 and BH10) were drilled to confirm the possible presence of the cavities indicated in the electrical resistivity survey. The location of boreholes is given in Figure 14.

**Figure 15:**
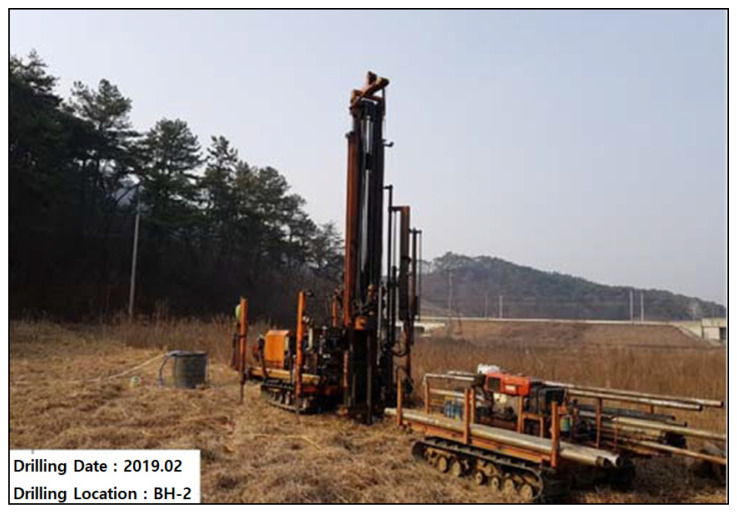
Rotary drilling rig using the NX-size core barrel (ϕ76 mm).

The borehole drilling depth was more than 50 m below ground level, to cover possible anomalies encountered in the geophysical resistivity survey. The drilling was performed using a self-propelled rotary drilling rig and a NX-size double core barrel (ϕ76 mm), as shown in Figure 15. The drilling rods used were generally 3 m long. Standard penetration testing (SPT) was carried out for indirect density measurement of alluvial and colluvial layers and completely weathered rock. Casing was installed in the upper part through the overburden and weathered rock to prevent borehole collapse. The groundwater level was monitored and recorded.

The extracted rock core was examined in detail to evaluate the rock characteristics, discontinuous distribution (spacing and dip), total core recovery (TCR), and rock quality designation (RQD), and this was measured and recorded in borehole logs.

**Figure 16:**
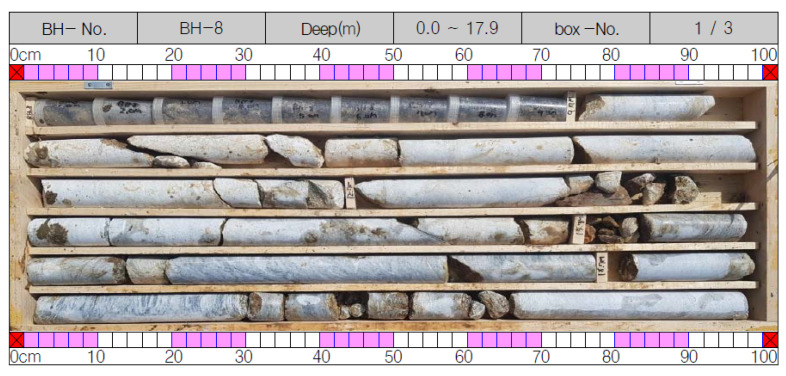
A core box from the present research.

A typical core box from this investigation is shown in Figure 16.

### 4.1. Borehole Investigation Results 

A total of 16 boreholes, which included two replacement holes, were drilled in the area during two drilling investigation campaigns. Standard penetration tests (SPT) were performed to assess the consolidation state of the alluvial overburden. Similarly, a borehole imaging processing system (BIPS) was used to inspect the borehole walls for cavities and fracture zones. BIPS images were recorded and correlated with core boxes when preparing the borehole logs.

Borehole data from these boreholes and laboratory tests performed on the selected core samples were reviewed and evaluated as part of this research. The main geological and geomechanical data are summarized in Table 3.

### 4.2. Bedrock Conditions

The bedrock conditions revealed by the first drilling investigation generally show competent to strong limestone. There is enhanced weathering and clayey infilling along the vertical to steeply inclined joints with striations and clay coating; however, the weathering effects decrease with depth, as expected. Similarly, the rock quality designation (RQD) values normally increased with depth, except in fracture zones and reflect the quality of drilling [25].

**Figure 17:**
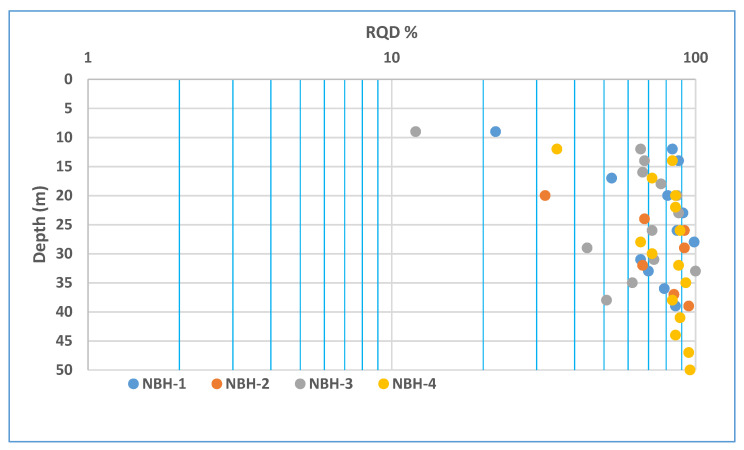
RQD values recorded in NBH-series boreholes.

RQD values for the NBH series boreholes drilled in 2014 indicate normal rock mass conditions, with RQD values reaching between 70 and 100% with increasing depth, as shown in Figure 17. This is normal and does not suggest the presence of cavities or zones of high weathering at depth.

**Figure 18:**
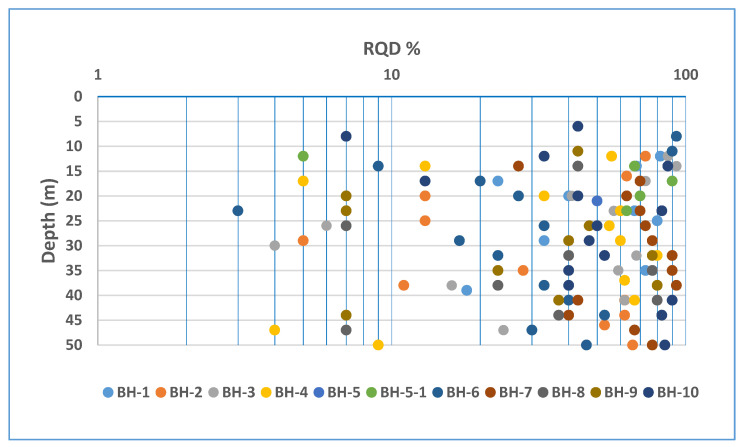
RQD values recorded in BH-series boreholes.

In comparison, the RQD values recorded in the 2019 drilling investigation in the adjacent area have a high scatter with no trend, as shown in Figure 18. The lack of trend indicates a poor drilling methodology and equipment use, resulting in the loss of core samples and grinding; thus, giving a relatively pessimistic impression, leading to a poor assessment of the rock mass conditions, which is not the case.

In addition, the inspection of core boxes from the 2019 drillings indicates better rock conditions of the recovered cores than the previous interpretation results. A reevaluation of the RQD values was not possible, due to the absence of location records of the core samples removed for laboratory testing.

The general rock mass conditions, as recorded at different levels of the mine, are generally good and consistent with boreholes NBH-1 to NBH-4. Table 4 summarizes the rock mass classification values, as recorded at various excavation faces at different mine levels.

Table 5 below, shows summary of characteristic parameters, the physical and mechanical properties of the limestone present in Gun-dong mine in the study area. The average uniaxial compressive strength of the limestone is around 81.4 MPa.

**Figure 19:**
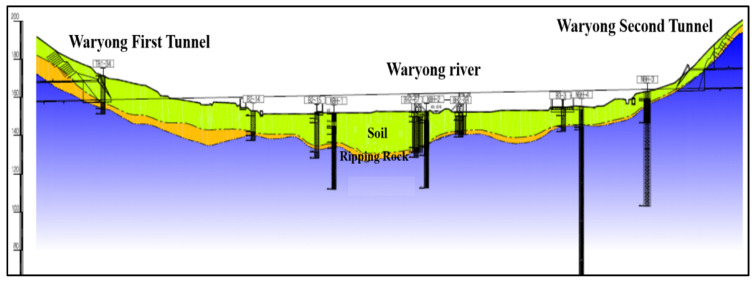
Longitudinal cross section along the railway embankment.

No significant cavities were recorded in the boreholes during the investigation of the high-speed railway by Korea Railroad Research Institute in 2016. Borehole logs prepared based on the recovered cores are comprehensive and provide a realistic picture of underground conditions along the railway embankment, as interpreted in the longitudinal section, and as given in Figure 19. 

The borehole drilling in 2018 consisted of 12 boreholes in farmland area. These included two replacement holes (BH-5A and BH-10A) to recover information lost due to the two abandoned boreholes mentioned above. Unfortunately, borehole collar elevations and coordinates were not recorded in borehole logs, making a precise reconstruction of the underground rock mass conditions difficult. 

**Figure 20:**
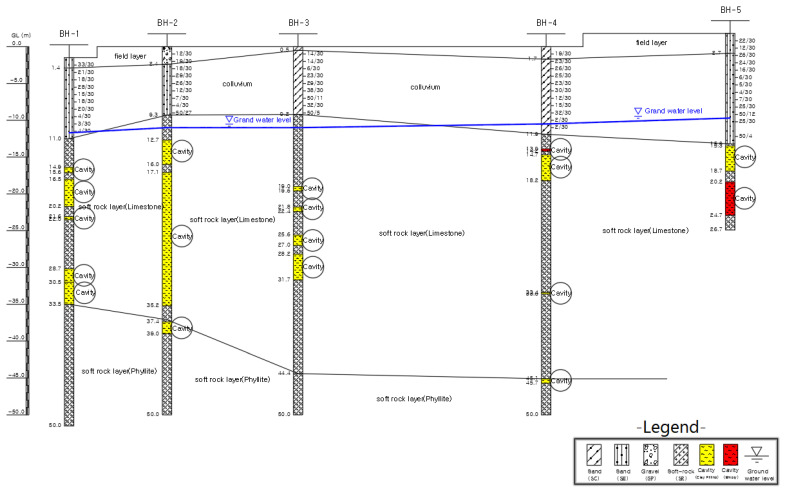
Developed geological section from 2018 drilling investigations (2019).

A geological section is given Figure 20. This section is the long line AA given in Figure 14, reproduced from the drilling report of 2018. The presence of large karstic cavities (up to 40 m across and 19 m high) indicated in this section are very unlikely, because of intercalated phyllite and limestone horizons.

**Figure 21:**
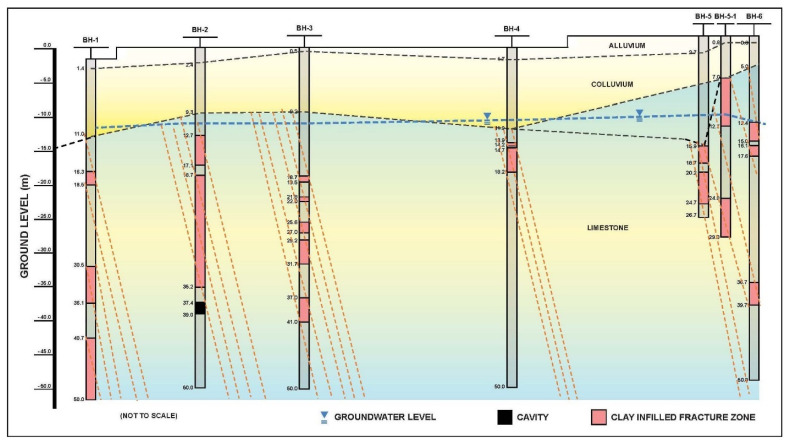
Reinterpreted geological section along BH−1 to BH−5.

The authors attempted to rework the available data and analyzed afresh the BIPS images and existing geological setting in the study area. The lack of borehole collar elevations and coordinates made precise interpretation quite difficult. Based on the authors’ reinterpretation and interpolation of the investigation data, a revised geological section is presented in Figure 21. This revised section is considered more representative of subsurface conditions, as it indicates the presence of steeply inclined highly weathered fracture zones, some of which are open, but without large cavities.

Borehole BH-5 was drilled near Sinkhole-3. By adding Boreholes BH 5-1 and BH-6 in the section, it appears that there is only limited deeply weathered zone in this area, due to a major joint set (N112E). There appears to be steep hydraulic gradients in the immediate proximity to BH-6, due to potential groundwater flow towards the Gun-dong mine. 

Figure 21 below shows the results of the reworked borehole investigation data and combined results of the geophysical and satellite imagery interpretation of available information. Instead of the previously postulated large cavities in the karstic rock mass, it is likely that vertical and sub-vertical joints and fracture zones along with sub-horizontal bedding joints reflect the rock mass conditions. The underlying bedrock had only a very minor role in the formation of the observed very shallow sink holes in the farmland. The main reason was lowering of the groundwater table and consolidation of colluvial and alluvial overburden.

### 4.3. Comments on the Borehole Drilling Investigation Quality

The core box photographs show slightly different rock conditions than those described in the borehole logs. This is due to the way core boxes were prepared and pessimistically interpreted. The main factors leading to the pessimistic interpretation may be summarized as follows:

1. Borehole elevations and coordinates are missing, making lateral interpolation very difficult.
2. The differentiation between colluvium and weathered rock is not always discernable.
3. Core-barrel and drilling bit type has not been identified, which largely determines the quality of the recovered cores and total core recovery.
4. There is no record of tool drops. Penetration rate is not given on the logs which could provide a good relation between the location fracture and soft zones.
5. There is no fracture log or description of infilled joints.
6. Drilling runs indicate that a 3-m long core-barrel was used, but borehole photographs in some cases show lack of spacers at correct locations making interpretation difficult.
7. Location of rock core samples, their condition, and length for laboratory testing has not preserved. Merely the length and location of samples has been marked on borehole logs.

In view of the above comments, the borehole investigation results are not conclusive and do not support the conclusions (presence of large-scale karst cavities) reached in the previously submitted reports.

### 4.4. Borehole Image Processing System (BIPS) Survey Data and Interpretation

**Figure 22:**
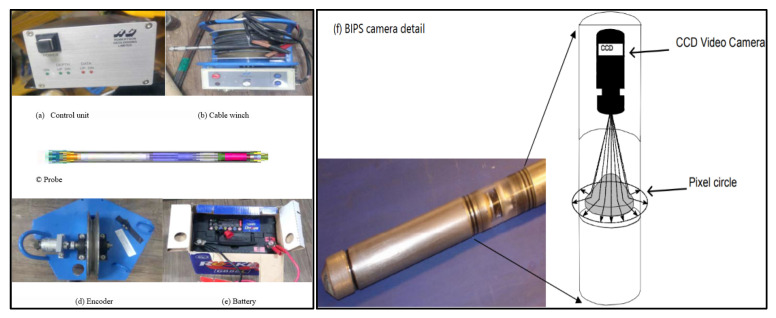
Main equipment accessories of BIPS system, (**a**) control unit, (**b**) cable winch, (**c**) probe, (**d**) depth encoder, (**e**) battery, (**f**) BIPS camera detail.

A borehole camera or borehole image processing system (BIPS) [26], when used in unlined boreholes, offers an excellent opportunity for direct and in situ inspection of open fissures, cavities, strata change, discontinuities, and joints. The main equipment and accessories of a BIP System are given in Figure 22. 

The system employs a video camera based illuminated probe, which provides high definition 360° images of the borehole wall. A closer inspection of a selected area is possible, to confirm the nature of the cavities in fracture zones. The inbuilt direction sensor enables ascertaining the dip direction of discontinuities and the alignment of open fissures and voids, etc. Careful surveying also provides valuable details on filling material, when analyzed in conjunction with the core boxes.

**Figure 23:**
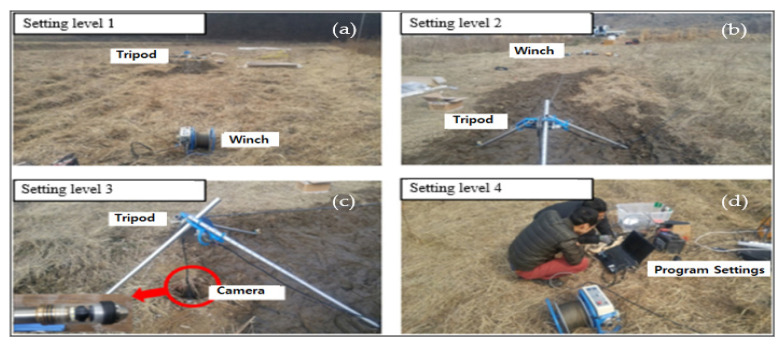
BIPS implementation in the study area, (**a**) Setting level 1, (**b**) Setting level 2, (**c**) Setting level 3, (**d**) Setting level 4.

The field deployment of the BIP system in the study area during the research work is shown in Figure 23.

Core samples collected through drilling investigation work can be supplemented by a borehole image processing system (BIPS) to compensate for the inherent limitations of drill hole data, where the orientation of discontinuities and opening of fractures is normally lost due erosion during drilling. 

### 4.5. The Use of BIPS in the Study Research

In this study, BIPS was used to identify from images, the discontinuities, cracks, and cavities of rocks and relate these to borehole cores.

**Figure 24:**
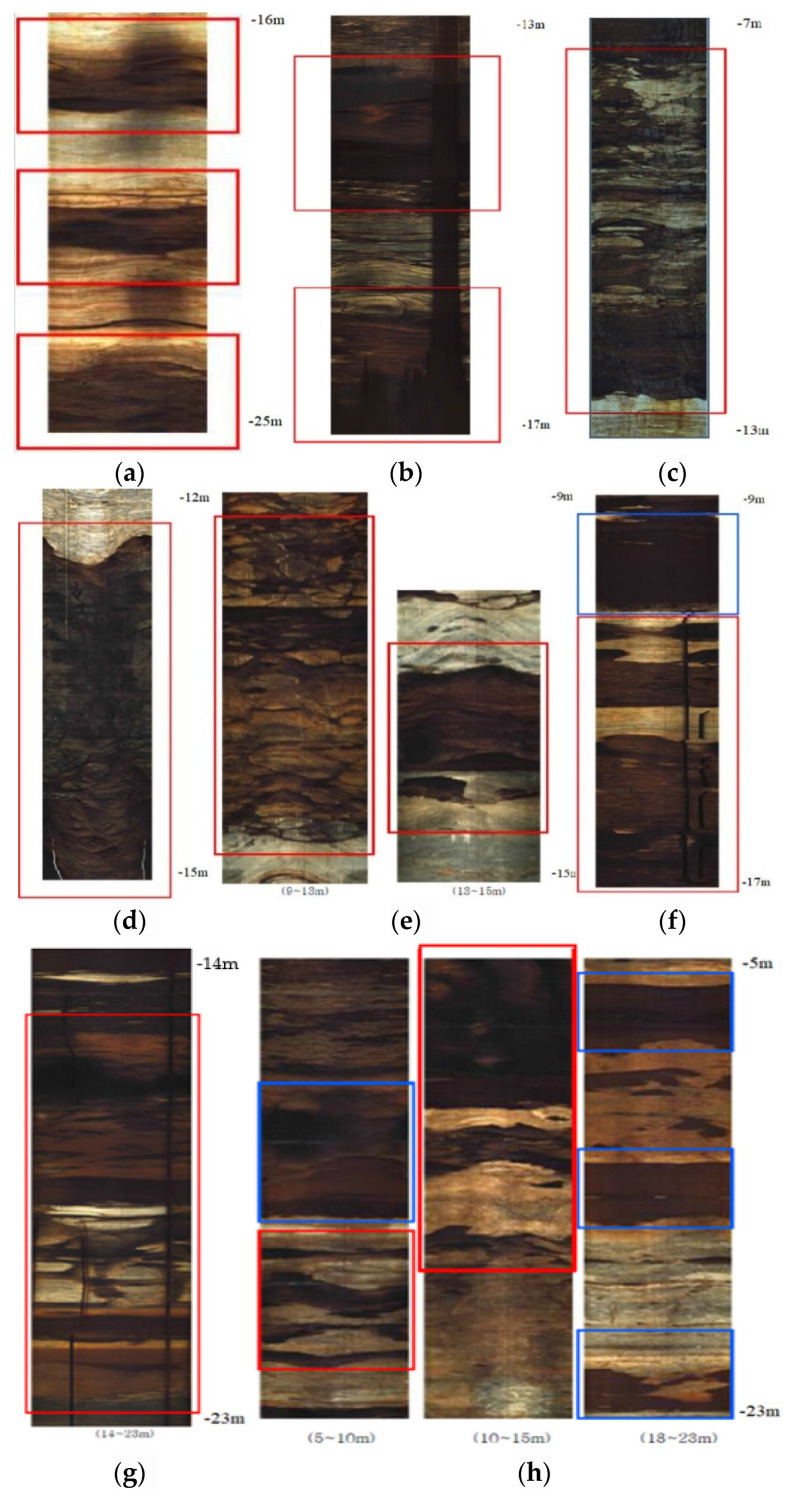
BIPS images showing clay filled fracture zones and potential cavities, (**a**) BH-3, (**b**) BH-4, (**c**) BH-5-1, (**d**) BH-6, (**e**) BH-7, (**f**) BH-8, (**g**) BH-9, (**h**) BH-10.

The following are the screenshots acquired using BIPS. The photographic data of the sections where potential cavities and fracture zones were identified has been correlated to the borehole drilling records, as shown in Figure 24. It can be noted that BH-1 and BH-2 boreholes were not surveyed by BIPS because of obstructions, due to a dropped PVC probing pipe.

Most of the available BIPS images show the presence of inclined fractures or joints and bedding joints with limited openings or thickness. Most of these are infilled with clayey silt material rock debris, including large rock fragments. In certain boreholes, there was a significant loss of cores which could be mistakenly considered as large fracture zones or an open cavity. However, the above BIPS images show this to be damage incurred and samples lost during drilling.

Notwithstanding the above, the BIPS scans are incomplete, as images lack directional orientation data, which could have supplemented and improved the understanding of underground rock mass conditions and the improvement in borehole log data. Loss of information is a reminder that trained staff familiar with investigation objectives should be deployed to obtain useful and meaningful results. The interpretation of BIPS images should be done at site using drilling information and recovered core samples. In return, the information, thus, obtained from visual inspection of BIPS images should be used for the enhancement and completion of borehole logs.

## 5. Hydrological and Hydrogeological Investigations

**Figure 25:**
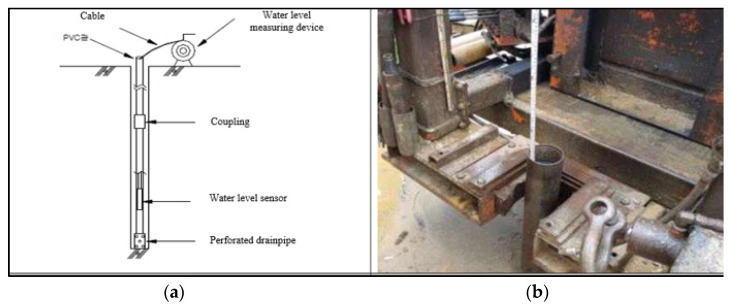
Measurement of groundwater level, (**a**) typical detail, (**b**) in the study area.

There are no historical groundwater monitoring data in the study area. During investigations, the depth from the borehole collar to groundwater levels was measured every 24 to 72 h in each borehole, after completion of the drilling operation. The equipment used is shown in Figure 25. Unfortunately, no piezometers were installed, which could have allowed monitoring of seasonal changes in local aquifer conditions over a long-term period. It should also be noted that variations in piezometric pressures with depth in open boreholes cannot be determined without fit for purpose piezometers. As such, potentially very useful information relevant to the sinkhole formation was lost. There is only a minor cost for piezometer installation once a borehole is in place. As such, normally all accessible boreholes should be fitted with piezometers for long-term monitoring. 

The method and equipment for the groundwater measurement used in the study area is shown in Figure 25. 

**Figure 26:**
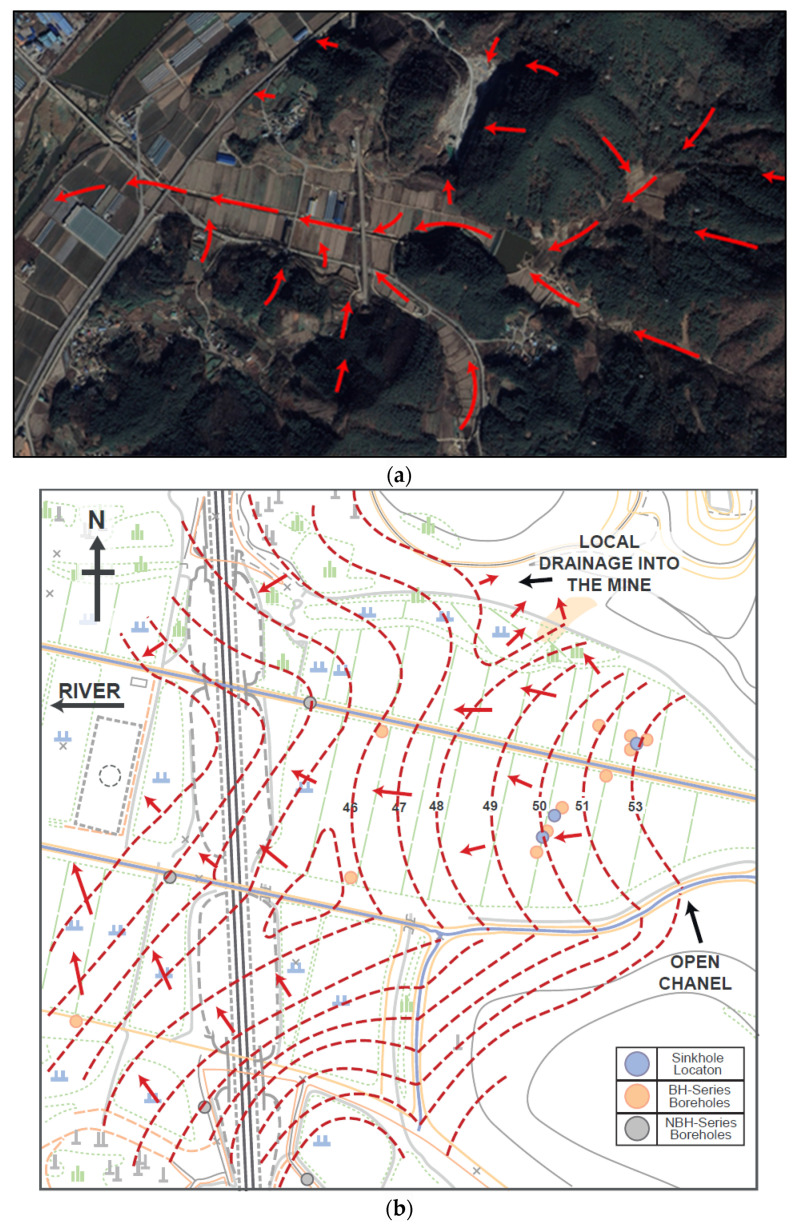
(**a**) Groundwater flow direction towards Hwangnyong river. Note the minor local inflow in the mine. There is no indication of any soil leaching, (**b**) interpreted groundwater flow direction-based groundwater contours. For base map information, see Figure 14.

Only a rough interpretation of the groundwater conditions can be made by short term monitoring of the boreholes. Based on this general pattern of groundwater flow, except in the immediate vicinity of the mine in borehole BH-6, piezometric contours show subsurface flow away from the mine in a south-easterly direction, towards the river (Figure 26).

### 5.1. Relationship between Precipitation and Mine Drainage

**Figure 27:**
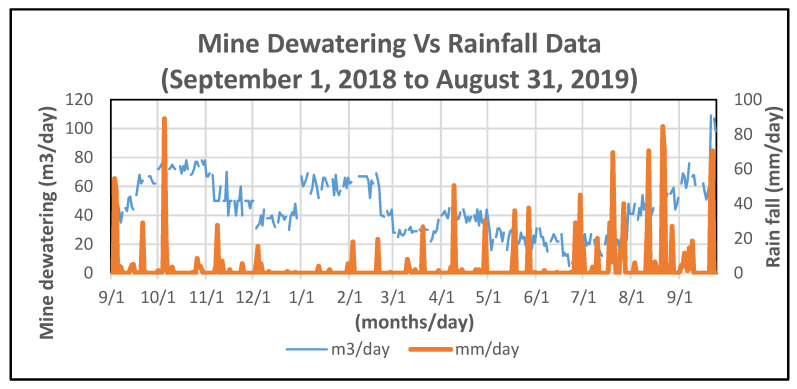
Monthly cumulative precipitation and discharge data (1 September 2018 to 31 August 2019).

In order to establish changes in the groundwater regime that may have led to sinkhole development, the water balance in the area was checked. Figure 27 shows cumulative precipitation in the Jangseong area and the pumped drainage water from Gun-dong mine. During the research period in this area, the annual precipitation was 1245 mm, slightly less than the annual average of 1283 mm. The water used in the Gun-dong mine is estimated to be 43 m^3^/day.

During this one-year period, there is a time-lagged correlation between rainfall and pumping discharge from the mine between March and November 2018, inclusive. However, there is no such correlation between December 2018 and February 2019, when there was high pumping discharge rate during a relatively dry period. The need for excess pumping may have been due to increased mining activity or operational reasons. 

It should be noted that the surface drainage was diverted away from the mine by constructing a dyke to prevent inundation or flooding of the mine. This prevents excessive water ingress into the mine.

**Figure 28:**
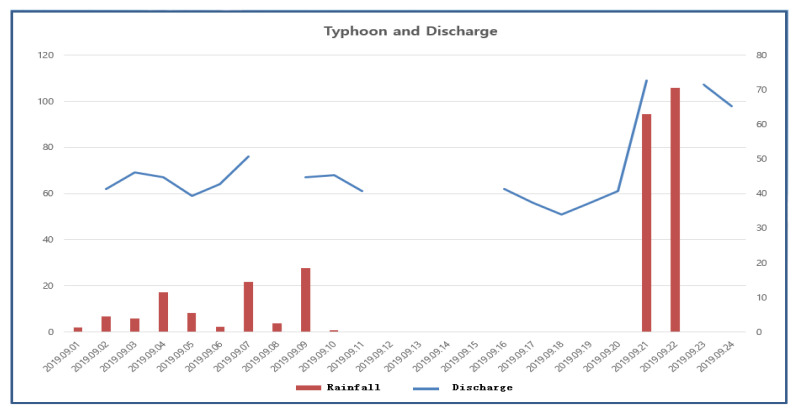
Increase in precipitation during typhoons.

There was an exception during typhoons. Typhoon Lingling brought rain and strong winds to the Jangseong area between 31 August and 7 September 2019. Heavy downpours occurred during the latter part, after the strong winds had died down. On the other hand, Tapa was a typhoon that hit the area between 21 and 22 September 2019 with heavy concentrated rains (>130 mm in 2 days). During this period, no pumping was done, due to the weekend and Chuseok holidays. However, there was high discharge from the mine during the Tapa downpour in the short term. Figure 28 shows the relationship between precipitation and pumping data of the Typhoons Lingling and Tapa.

### 5.2. Dewatering Arrangements in the Mine

**Figure 29:**
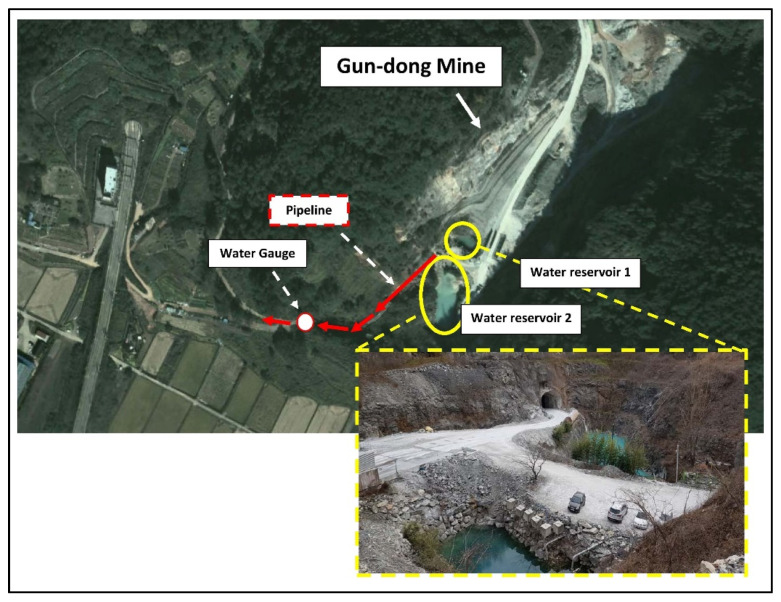
Drainage system of the Gun-dong mine.

Figure 29 shows the water supply and drainage arrangements in Gun-dong mine. The metered pumping discharge and V-notch measurement show the approximate amount of water being used in the mine. The wastewater of the mine is stored in a small reservoir, as shown. Water which is used in the lower levels of the mine is also stored and pumped out to the higher levels and used in mining activities. Residual water from the mine is pumped back to the reservoir situated just outside the first level of the mine. The drainage and pumping pipes are 150 mm in diameter. Additionally, and in case of heavy rain a 100 HP motor pump is used to pump excess water to a nearby tributary of the river.

## 6. Discussion 

Between 2014 and 2018 several sinkholes appeared in the farmland adjacent to Gun-dong mine. Investigations carried out in 2016 and 2018 were aimed at determining the stability of the railway line embankment in relation to the development of sinkholes, and potential damage the sinkholes posed for the farmland. 

The investigation involved the use of a combination of different methods suitable for sampling and determination of subsurface soils’ density, their composition, and underlying rock mass conditions. These included electrical resistivity profiling, borehole drilling, borehole camera imaging to establish geological cross sections. The detailed stability of the mine itself, with respect to the buckling of rock mass using finite element analysis, was also evaluated. This showed no notable settlement or buckling of rock cover over the limestone mine.

Full understanding of the existing geological and hydrogeological conditions, as well as thorough knowledge of rock mass conditions is essential for the determination of sinkhole causes and for the recommendation of remedial or mitigation measures. The interpretation of combined data by experienced personnel would have allowed a better understanding of the sinkholes’ appearance. 

The quality of borehole records from the second investigation campaign is somewhat poor and may have resulted in loss of potentially useful information. Considering that borehole collar elevations and coordinates are given, various depths mentioned about the boreholes lack accuracy. The same applies to groundwater levels.

All borehole scans obtained using a BIPS camera showed the presence of apparent fractures, ranging from open, to partially or fully filled with clay and rock debris. However, no open cavities of any significant dimensions were detected. The rock cores from the boreholes in the study area, as detailed in Figure 16, confirm the above conclusion. 

The standard penetration test (SPT) results indicate the extensive presence of unconsolidated layers with low SPT values. Densification of these areas, when combined with migration of fines from soils, due to lowering of the groundwater table, may have caused the formation of small-scale sinkholes, as observed in the farmland near the mine. 

## 7. Conclusions and Recommendations

1. Although official reports, because of the 2018 investigation, indirectly relate the development of sinkholes to interpreted karst cavities in limestone rock mass and nearby limestone mining for cement production, no direct relationship beyond doubt has been established. It should be noted that the first subsidence in the area was observed in 2008, before any significant underground mining activity in Gun-dong mine.
2. There is some evidence of groundwater level drops in the area, due to increased extraction for urban and agriculture use, as well as a discernable hydraulic gradient in the groundwater table, as documented in Figure 20 and Figure 21. This research paper concludes that the consolidation of overburden soils and lowering of the groundwater table due to increased water demand, in conjunction with minor migration fines are the possible causes of the formation of sinkholes.
3. Future extraction of groundwater and its use for the local area will result in further lowering of the groundwater table in the area, with a possible increased incidence of subsidence in the future. A comprehensive groundwater monitoring system can prove this trend and changes in aquifer conditions. This will require a well-planned network of piezometers for the determination of groundwater gradient and flow direction.
4. The appearance of sinkholes along a known joint set (N112 to 125E) orientation may have a link to the underlying karst fabric of the limestone rock mass. Moreover, their appearance, within a short time of each other may, allowing for a reasonable lag time, point to an increasing rate of groundwater depletion, due to increased water use.
5. Unfortunately, sinkholes were backfilled without collection of pertinent monitoring data, such as continued subsidence, soil profile determination, and appearance of further concentric cracks, etc. It is recommended to install and monitor geodetic survey elevation targets on steel rods in similar situations at the location of the sinkholes.
6. The future appearance of holes should be recorded, and these holes should be maintained untouched for monitoring purposes. Tracers may be used to determine preferential seepage paths, if any.
7. Disused mines make ideal storage or repositories for industrial waste. However, limestone mines with karst can lead to leaching and groundwater pollution. This should be avoided. In case it is decided to keep the mine open after its useful life, it is recommended that areas of the mine closer to farmland no longer in use should be backfilled with non-cohesive gravel or rock masonry debris and grouted to prevent settlement in the long term.
8. The formation of sinkholes reflects either a naturally changing environment or the result of human activities. A thorough long-term environmental risk assessment is needed for the formulation of necessary mitigation measures to limit adverse environmental and social impacts. This is particularly important when sinkholes appear in, or close to, human habitats.

## Figures and Tables

**Table 1:** Regional stratigraphic data.

Geological Age	Lithology
Cenozoic Quaternary	Alluvium (Qa)
Mesozoic Cretaceous Period	Granite intrusive rocks and acid dykes (Kad and Kgr)
	Neutral and basic volcanic rocks (Kiv)
	Jinan formation sandstone, mudstone (Knis, Knas)
Unknown	Intrusive rocks granites (Jgr), amphibolite (Jdi), foliated granites (Jpgr),
Jurassic and Mesozoic	Layer group lower phyllite (Og2), limestone (ls), quartzite (q)
Precambrian	Sobaeksan Henhor and granitic gneiss complex (PCEggn)

**Table 2:** Mineral content of Okcheondae limestone.

Limestone Mineral Content	Percentage (%)
Calcite	69.7
Sericite (Muscovite and Illite)	10.1
Pyroxene	7.8
Quartz	5.3
Plagioclase	3.5
Amphibole (openwork)	3.2
Others	0.4
Total	100%

**Table 3:** Summary of lithological data obtained from the investigation boreholes.

Estimated Thickness (Approx. below El. 61 m)	Lithological Unit	Description
0.5 m to 2.8 m	Alluvium	The topsoil and alluvium are dark brown and grayish brown in color, and composed of sand mixed with clay, silty sand, and sand mixed with gravel. It has normal density (N:12/30 cm~33/30 cm).
3.1 m to 12.3 m	Colluvium	Colluvium is comprised of dark brown mixed sand and with clayey silt pockets and discontinuous layers. It is very loose in places to very dense (N = 2/30 cm~50/4 cm).
25 m to 240 m	Limestone	The main horizon in the upper section is dolomitic and calcareous limestone.
Unknown	Phyllites	Limestone is underlain by dark brown phyllites.

**Table 4:** Summary of Rock Mass Classification Values.

Mine Level Number	Approx. Elevation (m asl)	Number of Excavation Faces Logged	Average Rock Mass Classification Values		
			RMR	GSI	Q-Number
Levels 1 to 2	16/−4	Records not available			
Level-3	−24	7	64	70	10.5
Level-4	−44	12	68	71	17.7
Level-5	−64	13	71	75	24.0
Level-6	−84	11	70	75	37.5
Level-7	−104	13	70	74	30.0
Level-8	−124	16	62	66	13.3
Level-9	−144	4	70	71	29.0

**Table 5:** Mechanical Properties of Limestone Present in the Study Area.

No.	Unit Weight (kN/m^3^)	Specific Gravity	Elastic Wave Velocity (m/s)	Tensile Strength (MPa)	Compressive Strength (MPa)	Modulus of Elasticity (GPa)	Poisson’s Ratio (υ)
1	26.2	2.70	4530	8.0	85.9	47.9	0.24
2	26.6	2.70	5540	8.3	74.7	53.5	0.28
3	26.9	2.72	5580	9.2	83.6	56.7	0.26
Avg	26.6	2.71	5220	8.5	81.4	52.7	0.26

## Data Availability

The data presented in this study are available on request from the corresponding author.
